# Naming Ability Changes in Physiological and Pathological Aging

**DOI:** 10.3389/fnins.2012.00120

**Published:** 2012-08-20

**Authors:** Maria Cotelli, Rosa Manenti, Michela Brambilla, Orazio Zanetti, Carlo Miniussi

**Affiliations:** ^1^IRCCS Centro San Giovanni di Dio FatebenefratelliBrescia, Italy; ^2^Department of Clinical and Experimental Sciences, National Neuroscience Institute, University of BresciaBrescia, Italy

**Keywords:** language, brain stimulation, HAROLD, plasticity, cognition

## Abstract

Over the last two decades, age-related anatomical and functional brain changes have been characterized by evidence acquired primarily by means of non-invasive functional neuroimaging. These functional changes are believed to favor positive reorganization driven by adaptations to system changes as compensation for cognitive decline. These functional modifications have been linked to residual brain plasticity mechanisms, suggesting that all areas of the brain remain plastic during physiological and pathological aging. A technique that can be used to investigate changes in physiological and pathological aging is non-invasive brain stimulation (NIBS). The present paper reviews studies that have applied NIBS in younger and older adults and in patients with dementia to track changes in the cerebral areas involved in a language task (naming). The results of this research suggest that the left frontal and temporal areas are crucial during naming. Moreover, it is suggested that in older adults and patients with dementia, the right prefrontal cortex is also engaged during naming tasks, and naming performance correlates with age and/or the degree of the pathological process. Potential theories underlying the bilateral involvement of the prefrontal cortex are discussed, and the relationship between the bilateral engagement of the prefrontal cortex and the age or degree of pathology is explored.

One of the primary areas of investigation in neuroscience is **age-related brain changes** and the associations between these changes and changes in cognitive (e.g., language) function. Progress regarding the study of physiological and pathological brain aging achieved over the last two decades has provided strong evidence of neurophysiological correlates of cognitive and behavioral changes associated with aging.

For example, studies at the neuronal level have demonstrated that dopaminergic decline and gray matter atrophy are both correlated with specific cognitive changes in older adults (Brody, 1955; Coleman and Flood, 1987; West et al., 1994; Small et al., 2002; Resnick et al., 2003; Sowell et al., 2003; Burke and Barnes, 2006; Kramer et al., 2007). In addition, it has been clearly demonstrated that processing speed, memory, and executive functions depend on the “well-being” of several neuronal substrates (Kennedy and Raz, 2009). Structural imaging results have also demonstrated widespread gray and white matter tissue atrophy, which largely occurs in the frontal cortex (e.g., Raz et al., 2005).

Based on evidence primarily acquired via non-invasive functional neuroimaging, four principal hypotheses have been postulated to explain the relationship between age-related neuronal activity changes and cognitive performance (Reuter-Lorenz and Park, 2010). First, an overactivation of some cortical areas and a reduction in the hemispheric asymmetry of activation has been documented in older adults as compared to younger adults during cognitive task execution (Grady et al., 1992; Backman et al., 1997; Cabeza, 2002; Park and Reuter-Lorenz, 2009). This frontal lobe overactivation has been reported in episodic memory, working memory, and perceptual tasks (Grady et al., 1995, 1998; Cabeza, 2002; Grady, 2008). By reviewing age-related differences in prefrontal cortex activity during working memory and episodic memory tasks, Rajah and D'Esposito (2005) reported both age-related decreases and increases of activity in specific prefrontal regions.

Second, a loss of regional specialization or declining specificity, referred to as dedifferentiation, has been hypothesized to occur in older adults (Reinert, 1970; Lindenberger and Baltes, 1994; Chee et al., 2006; Voss et al., 2008). Ventral-visual activity in older adults is characterized by the dedifferentiation of responses to different stimuli categories, such as faces and/or houses (Park et al., 2004). In addition, Goh et al. (2010) reported that the dedifferentiation of neural responses in older adults is associated with a reduction of the distinctiveness of within-category representations in the ventral-visual cortex.

Third, frontal compensation has been investigated in older adults because higher prefrontal activation is more prevalent in older adults than in younger adults during several cognitive tasks (Cabeza et al., 2003; Gutchess et al., 2005; Davis et al., 2008; Heuninckx et al., 2008; Eyler et al., 2011; Cabeza and Dennis, in press). Neuroimaging studies have revealed an age-related reduction in occipito-temporal activity coupled with an increase in frontal activity, a pattern referred to as the posterior-anterior shift in aging (Davis et al., 2008).

Fourth, the default network theory postulates that the activity in several regions of the default mode network is altered during the execution of several cognitive tasks; these regions includes the medial prefrontal cortex and the medial and lateral parietal cortex (Raichle et al., 2001; Lustig et al., 2003; Reuter-Lorenz and Lustig, 2005; Persson et al., 2007; Miller et al., 2008; Mevel et al., 2011). Furthermore, the activity in the major components of the default mode network remains stable in healthy, older individuals, whereas the activity in a number of discrete cortical areas located in the prefrontal, temporal, and occipital regions changes over time (Beason-Held et al., 2009). Indeed, all of these hypotheses largely overlap, but all of them at least partially underline the cerebral tissue's ability to change its structure and function continuously in response to environmental demands.

All of these physiological-aging-induced structural and functional changes have been linked to residual brain plasticity to counteract neural loss (Jancke, 2009). It has therefore been suggested that neural plasticity facilitates alternative “**neural strategies**” to maintain an adequate level of cognitive performance (Greenwood, 2007; Zollig and Eschen, 2009). The scaffolding theory of aging and cognition (STAC; Park and Reuter-Lorenz, 2009) presents a reunified vision of the dynamic brain changes that occur in response to naturally occurring functional alterations across the life span. According to the STAC, the brain responds to physiological aging by forging alternative brain circuitry (scaffolds); although they are less efficient, this process permits the individual to maintain a high level of cognitive functioning. Consequently, brain scaffolding would result in a pattern of overactivation and eventually reduced lateralization, which is consistent with the results of previous neuroimaging studies; however, this pattern of overactivation would be present in the frontal cortex and in the parietal, medio-temporal, and occipital regions (Reuter-Lorenz and Park, 2010).

## Neural Correlates of Object and Action Naming

Language skills are examples of cognitive abilities that change during aging. Human language is a complex behavior that involves multiple processes (Pulvermuller, 2003). Similar to other functional abilities, language processing has been shown to be lateralized to brain regions of the left hemisphere. One important process in the constellation of language skills is naming. Evidence from both lesion and imaging studies suggests a central role of the left prefrontal, temporal, and parietal areas during naming, although some involvement differences exist for object (noun) versus action (verb) naming (Daniele et al., 1994; Perani et al., 1999; Price et al., 2005). Naming is an ability that shows adaptation during aging; however, few studies have investigated how the engagement of these areas changes during the performance of naming tasks in older adults.

In brain-damaged patients with acquired aphasia, selective category-specific deficits have been described for grammatical word classes, such as for nouns and verbs (Miceli et al., 1984). Several clinical observations have suggested that different cerebral areas are involved in noun and verb processing. Ample evidence suggests that aphasic patients may be selectively impaired in object naming but not in action naming or vice versa (Miceli et al., 1984; Baxter and Warrington, 1985; McCarthy and Warrington, 1985; Miceli, 1988; Miceli and Caramazza, 1988; Caramazza and Hillis, 1991; Miozzo et al., 1994). Patients with a selective disorder for object naming typically have lesions localized to the left temporal lobe; conversely, a selective impairment in action naming has been associated with larger lesions, which typically extend to the left frontal cortex (Daniele et al., 1994).

In conclusion, various studies have convincingly demonstrated that the lexical system is organized according to grammatical class. Furthermore, Damasio and Damasio (1992) suggested that mediation systems for verbs may be located in frontal and parietal sites.

These results were considered to support the findings of focal lesion studies that suggested the frontal lobe plays a central role in verb processing (Cappa and Perani, 2003; Shapiro and Caramazza, 2003a,b; Silveri and Ciccarelli, 2007). Functional brain imaging studies involving patients and neurotypical participants have provided evidence for the selective recruitment of brain areas associated with noun and verb processing (Perani et al., 1999; Shapiro et al., 2006). Specifically, actions apparently evoke stronger activation than objects in the bilateral posterior middle temporal cortex, in the left temporo-parietal junction, and in the left frontal cortex (Liljestrom et al., 2008). Nevertheless, recent studies have suggested that the relationship between the grammatical class and the related pattern of brain activation is not clear-cut and must be more thoroughly investigated (Pulvermuller et al., 1999, 2012; Crepaldi et al., 2011; Vigliocco et al., 2011).

## Naming in Pathological Aging

The study of degenerative conditions has also provided converging evidence; specifically, a severe impairment in action naming has been identified in patients with frontal dementia (Cappa et al., 1998). The diagnostic label of frontotemporal dementia (FTD) encompasses a number of heterogeneous clinical presentations, in which different patterns of neuropsychological impairment in linguistic processing, executive function, and action organization reflect the location of the underlying pathology. Cotelli et al. (2006a) reported that action naming is impaired in comparison to object naming in patients with FTD, and some differences exist between different FTD variants. A severe action naming disorder has been observed in patients with Non-fluent Primary Progressive Aphasia (NfPPA), Progressive Supranuclear Palsy (PSP), and Corticobasal Degeneration (CBD), while no significant difference in object and action naming was demonstrated in Semantic Dementia (SD; a subtype of FTD associated with higher temporal atrophy localization). Overall, these findings confirm the crucial role of the frontal cortex in action naming but not in object naming. Furthermore, the observation of a severe impairment in verb retrieval in patients with NfPPA is not unexpected. This variant is associated with a clinical presentation similar to Broca's aphasia that reflects prominent pathological involvement of the anterior language areas (Hillis et al., 2004). The results were less expected in the case of PSP and, in particular, of CBD because a verb-naming disorder in PSP had originally been reported (Daniele et al., 1994; Bak et al., 2001).

The finding of a severe verb production deficit in two conditions that are clinically characterized by a prominent movement disorder implicates a link between action-related language and action representation (Hauk et al., 2004; Tettamanti et al., 2005). In another report, Cotelli et al. (2007) described a particularly severe impairment in action naming associated with Parkinson disease (PD). PD is a neurodegenerative process characterized by several motor and cognitive clinical manifestations for which effective, mechanism based treatments remain elusive (Rodriguez-Oroz et al., 2009). Compared to the controls, PD patients showed a deficit both in action and object naming. In addition, PD patients were significantly more impaired in action naming than in object naming; there was a significant positive correlation between the severity of the action naming impairment (and the degree of action-object naming dissociation) and the severity of the visual and verbal long-term memory impairments, which are the classical features of PD neuropsychological impairment. The severity of the verb retrieval impairment in PD patients may be a consequence of dopamine depletion in the striatum, which would disrupt the function of the subcortical prefrontal networks (Alexander et al., 1986). These results may be due to an initial executive impairment (Muslimovic et al., 2005; Zgaljardic et al., 2006) or a specific language dysfunction. Recently, an fMRI study concluded that frontal-motor dysfunction in PD affects tasks that require a complicated action-related search (Peran et al., 2009), suggesting the presence of a linguistic dysfunction in these patients. Further investigation of these conditions may provide additional insight into the relationships between the localization of cortical involvement, the pattern of lexical impairment, and the specific features of high-order motor dysfunction experienced.

These findings are particularly interesting given the brain modifications that are associated with physiological aging. When interpreting the dissociation of action and object processing in older adults, one should consider the role of the frontal cortex and the dynamic alteration of this area due to aging. These changes are reflected in functional compensation processes; if the STAC theory is correct, these compensation processes should be associated with specific changes in naming performance.

## Naming and Healthy Aging

Some important domains of cognitive ability decline with age, including processing speed, memory, reasoning, and language (Salthouse, 2010). In general, the effect of normal aging on language is characterized by a complex pattern. Performance on comprehension and vocabulary knowledge is well maintained (Burke and Shafto, 2008; Shafto et al., 2009; Tyler et al., 2010), while word finding performance declines (Albert et al., 1988; Goral et al., 2007; Wierenga et al., 2008). A progressive reduction in lexical retrieval performance has been well-documented in older adults (Nicholas et al., 1985; Bowles et al., 1987; Albert et al., 1988; Au et al., 1995; Barresi et al., 2000; Mackay et al., 2002; Morrison et al., 2003; Connor et al., 2004; Mortensen et al., 2006). Unlike the retrieval of word meaning, which appears to be preserved and even enhanced with aging (Verhaeghen, 2003; Goral et al., 2007), the ability to retrieve the sound or phonology of words seems to decline in older adults. People of all ages struggle to identify the correct word on a daily basis. This difficulty, referred to as tip-of-the-tongue phenomenon, is a temporary inability to access a word's phonology following a successful activation of semantic information (Cross and Burke, 2004; Schwartz and Metcalfe, 2011). Nevertheless, the frequency of tip-of-the-tongue experiences significantly increases with aging, and older adults report the inability to produce well-known words as one of the most annoying cognitive “symptoms” they experience (Burke and Shafto, 2004; Shafto et al., 2007). Recent neuroimaging findings have demonstrated that age-related word retrieval difficulties are associated with atrophy in linguistic areas and are correlated with white matter integrity across a broad range of regions that have been implicated in language production (Stamatakis et al., 2005, 2011). The frequency of tip-of-the-tongue episodes has been shown to be positively correlated with adult age and negatively correlated with gray matter density in the left insula (Shafto et al., 2007, 2009). Several other factors have been demonstrated to contribute to this decline, including gender (Ross et al., 1995), education (Goral et al., 2007), and general health status (Albert et al., 2009).

One of the primary paradigms used to evaluate word finding skills in the normal aging population has been the picture-naming test. Picture-naming is a complex task and a variety of issues could contribute to poor performance on this task, including visual problems, semantic deficits or grammatical deficits, loss of word forms, or impaired access to phonological forms (Price et al., 2005; DeLeon et al., 2007). Picture-naming is known to engage large and distinct neural networks (Price et al., 2005; Cotelli et al., 2006a) that are differentially required for the different processes underlying picture-naming (DeLeon et al., 2007; Liljestrom et al., 2008), and these networks have been demonstrated to undergo modification during aging (Wierenga et al., 2008).

Several studies have demonstrated an **age-related decline in object and action naming** (Goodglass, 1980; Nicholas et al., 1985; LaBarge et al., 1986; Ardilla and Rosselli, 1989; Feyereisen, 1997), suggesting that word retrieval, rather than lexical and semantic knowledge, declines in healthy aging (Goral et al., 2007). Some differences in object and action naming have been demonstrated in elderly individuals with controversial results (Nicholas et al., 1985; Barresi et al., 2000).

Differences in the performances of younger and older adult participants on a picture-naming task have also been related to a slowing of processing speed with age (Cotelli et al., 2010). In this respect, a number of studies have provided data consistent with the hypothesis that older adults are slower than younger adults when performing picture-naming tasks (Thomas et al., 1977; Mitchell, 1989; Morrison et al., 2003). Several studies have compared latencies between object and action naming in younger and older adult individuals, and they revealed that action naming is more difficult than object naming, both in terms of accuracy and in terms of latencies (Szekely et al., 2005; Druks et al., 2006).

**Figure 1:**
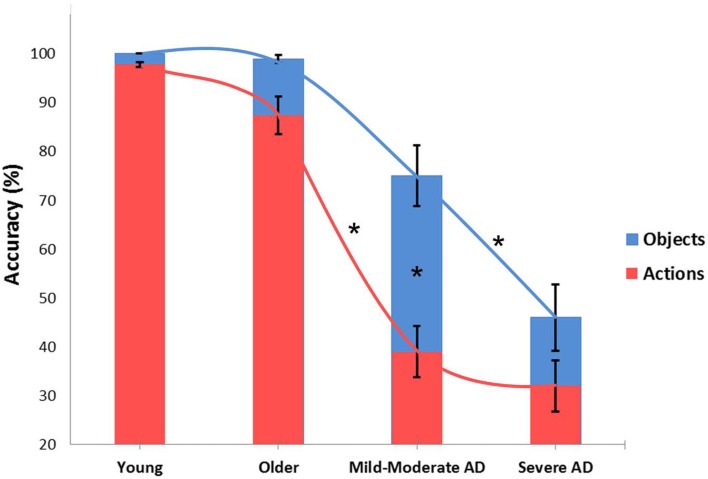
**Accuracy performance for object and action naming in young adults (9 participants; Cappa et al., 2002), older adults (13 participants; Cotelli et al., 2010), Alzheimer's Disease patients at mild to moderate stages of cognitive impairment (12 participants) and Alzheimer's Disease patients at severe stages (12 participants; Cotelli et al., 2008)**. Asterisks indicate significant effects (*p* < 0.05). Mild-moderate AD patients demonstrated significantly worse action naming accuracy than older adults and better performance in object naming than severe AD. Finally, a significant difference between action and object naming performance was only evident in the mild to moderate AD group. Errors bars indicate mean standard error.

**Figure 2:**
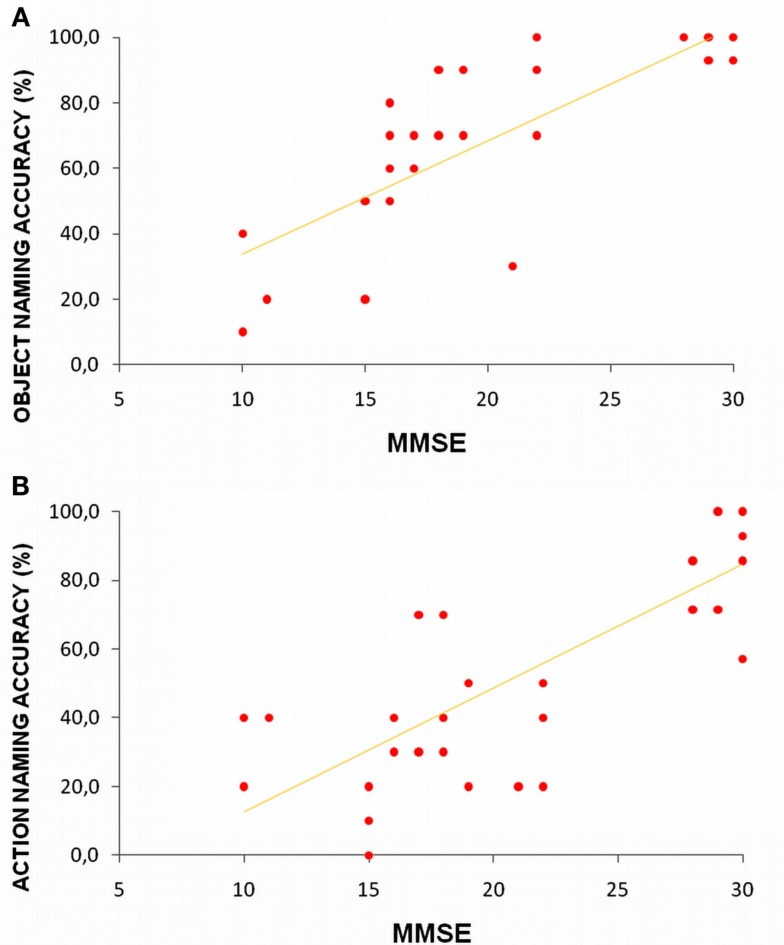
**Correlation between mini mental state examination (MMSE) and object (A) or action (B) naming performance**. Older adults and AD patients with mild to moderate or severe cognitive impairment are included.

Figure 1 reflects data collected in our laboratory and illustrates action and object naming correctness trends in younger and older adults and in Alzheimer disease (AD) participants at a different impairment stages (Cappa et al., 2002; Cotelli et al., 2006b, 2008, 2010). We performed an ANOVA (groups: younger, older, mild AD, severe AD) within-subjects (stimulus: object, action). This analysis revealed a significant effect between the stimulus and group [*F*(3, 40)  = 8.052, *p*  = 0.0003]. *Post hoc* analyses (Bonferroni) were conducted. As shown in Figure 1, small differences in action naming performance were found between younger and older adult participants, but the differences were not statistically significant (Cappa et al., 2002; Cotelli et al., 2010). Moreover, patients in the early stages of AD (mild to moderate) demonstrated dramatically reduced action naming accuracy (*p*  = 0.000004), with a minor and insignificant decrease (*p*  = 0.08) in object naming performance compared to older adults. Finally, AD patients in the advanced stages of cognitive decline (severe) also demonstrated a drastic decrease in object naming accuracy (*p*  = 0.014; Cotelli et al., 2006b, 2008). These data suggest that there a continuum in the decline of picture-naming ability exists, and it correlates with physiological and pathological aging. A significant difference between action and object naming performance (i.e., higher accuracy for objects) was only evident in the mild AD group (*p*  = 0.000001). To investigate the possible effects of participant characteristics on naming performance, we performed a correlational analysis between age and naming abilities in all four groups, and we performed a similar analysis between mini mental state examination (i.e., cognitive impairment) and naming abilities in older participants and AD patients. Significant correlations between age and both object and action naming abilities (object naming: *R*  = −0.52, *p* < 0.001; action naming: *R*  = −0.65, *p* < 0.001) indicate a progressive decrease of naming abilities during aging. Furthermore, the significant correlations between mini mental state examination and both object and action naming accuracies (object naming: *R*  = 0.83, *p* < 0.001; action naming: *R*  = 0.81, *p* < 0.001) indicate that a progressive decrease of naming abilities is linked to cognitive deterioration (see Figure 2). Identifying the neural correlates of these picture-naming performance changes would further increase our understanding of the processes underling aging, and picture-naming may be important as a tool to evaluate the degree of aging-associated cognitive deterioration.

## Non-Invasive Brain Stimulation Studies on Naming in Younger Adults, Older Adults, and Dementia Patients

Since the beginning of the century, important technological advancements have occurred, which have allowed us to study the structures and mechanisms underlying cognitive functions in the human brain. One such development has been the introduction of **non-invasive brain stimulation (NIBS)**. NIBS approaches aim to induce changes in brain activity, which can lead to a wide range of behavioral alterations (e.g., Nitsche et al., 2008; Sandrini et al., 2011). NIBS techniques include transcranial magnetic stimulation (TMS) and transcranial direct current stimulation (tDCS). TMS and tDCS transiently influence behavior by altering neuronal activity through different mechanisms (Miniussi et al., 2010) which may have facilitative or inhibitory effects. The relevance of NIBS to cognitive neuroscience is primarily derived from its ability to transiently probe the functions of the stimulated cortical area/network, whereas its relevance to neurorehabilitation is derived from its ability to modulate cortical excitability (Cotelli et al., 2011a,b, 2012; Miniussi and Rossini, 2011). Several recent studies involving young adult participants, have reported improvements in picture-naming abilities following TMS administration (Table 1). Topper et al. (1998) applied single pulse TMS over one of three locations (left Wernicke's area, its right contralateral homologous or the left motor cortex) at different timings during an object naming task. The results demonstrated that TMS over Wernicke's area induced shorter reaction times (RTs) only if the TMS pulse was delivered 500–1000 ms prior to picture presentation. Subsequently, Mottaghy et al. (1999, 2006) and Sparing et al. (2001) confirmed the involvement of Wernicke's area in object naming using single pulse TMS and repetitive TMS (rTMS). Subsequent studies demonstrated that the temporal pole (Pobric et al., 2007) and middle temporal gyrus (Acheson et al., 2011) also play important roles in object naming. The inferior frontal cortex (in particular, Broca's area) has also been shown to be relevant during object naming. By applying three-pulse TMS over Broca's area, Schuhmann et al. (2009) showed an increase in RTs, and the same result was observed by Chouinard et al., (2009) using rTMS. Recently, Schuhmann et al. (2012) performed a chronometry study, and by delivering TMS at different intervals, they demonstrated that the temporal and inferior frontal areas are critical to naming ability. Schuhmann et al. (2012) demonstrated that the left middle temporal gyrus becomes functionally relevant 225 ms after picture onset; this result is followed by Broca's area at 300 ms and Wernicke's area at 400 ms. These results clarified and summarized the role of the inferior frontal cortex and temporal areas during naming.

**Table 1:** **Non-invasive brain stimulation studies on naming in young adults, older adults, and dementia patients**.

Study	N	Stimulation technique	Target area	Stimulated task, timing (ms, picture presentation – stimulation)	Results
TRANSCRANIAL MAGNETIC STIMULATION					
Topper et al. (1998)	65 HY	spTMS, 55% of SO	None (*N* = 10), left motor cortex (*N* = 15), WA (*N* = 35), WAH (*N* = 5)	Object naming, −5000/+300	TMS over WA at −500 or −1000 ms: ↓RTs
Mottaghy et al. (1999)	15 HY	On-line rTMS (20 Hz, 2 s), 55% of SO	WA, WAH, BA, primary visual cortex, placebo	Object naming, after rTMS, −120,000	TMS over WA immediately before picture: ↓RTs
Sparing et al. (2001)	16 HY	On-line rTMS (1 Hz, 40 s), 55% of SO in 10 HY; on-line rTMS (20 Hz, 2 s), 35, 45, or 55% of SO in 6 HY	WA, BA, primary visual cortex, placebo	Object naming, after rTMS, −120,000	TMS over WA immediately before picture at 55% of stimulator output: ↓RTs
Cappa et al. (2002)	9 HY	On-line rTMS (20 Hz, 500 ms), 90% of MT	Right DLPFC, left DLPFC, placebo	Naming of objects and actions, 0	TMS over left DLPFC during action naming: ↓RTs
Mottaghy et al. (2006)	44 HY (spTMS), 36 HY (rTMS)	spTMS, 35–95% of SO; on-line rTMS (20 Hz, 2 s), 55% of SO	Left motor cortex (sp TMS), primary visual cortex (rTMS), WA (sp TMS, rTMS), WAH (sp TMS, rTMS), BA (rTMS), no stimulation (sp TMS), placebo (sp TMS, rTMS)	Object naming, spTMS: −3000/+300; rTMS: after rTMS, −120,000	spTMS over WA at −1000 and −500 with low intensities: ↓RTs rTMS over WA immediately after picture: ↓RTs
Cotelli et al. (2006b)	15 AD	On-line rTMS (20 Hz, 500 ms), 90% of MT	Right DLPFC, left DLPFC, placebo	Naming of objects and actions, 0	TMS over left and right DLPFC: ↑ correctness for actions
Pobric et al., (2007)	12 HY	Off-line rTMS (1 Hz, 10 min), 120% of MT	Temporal pole, no stimulation	Basic object naming, specific semantic category object naming, number naming, synonym judgment, number judgment	rTMS over Temporal pole: ↓RTs for specific naming; ↓RTs for synonym judgment
Cotelli et al. (2008)	24 AD (12 mild, 12 moderate to severe	On-line rTMS (20 Hz, 500 ms), 90% of MT	Right DLPFC, left DLPFC, placebo	Naming of objects and actions, 0	TMS over left and right DLPFC: ↑ correctness for actions in mild AD, ↑ correctness for actions and objects in moderate to severe AD
Schuhmann et al. (2009)	12 HY	tpTMS (40 Hz, 50 ms), 120% of MT	BA, placebo, no stimulation	Object naming, +150/+525	rTMS over BA at 300 ms: ↑RTs
Chouinard et al. (2009)	24 HY (12 object naming, 12 other experiments)	on-line rTMS (10 Hz, 400 ms), 90% of MT	Left and right lateral-occipital cortex (object naming, color naming, size discrimination), left and right IFG (object naming, color naming, size discrimination, reading, and categorization), placebo (object naming)	Object naming, color naming, size discrimination, reading, and categorization, 0	rTMS over lateral-occipital cortex: ↑RTs of object naming and size judgment in contralateral spaces, rTMS over left IFG: ↑RTs of object naming in both spaces
Cotelli et al. (2010)	30 HE	On-line rTMS (20 Hz, 500 ms), 90% of MT	Right DLPFC, left DLPFC, placebo	Naming of objects and actions, 0	TMS over left and right DLPFC: ↓RTs for actions
Acheson et al. (2011)	14 HY	On-line rTMS (10 Hz, 400 ms), 110% of MT	Left STG, left MTG, no stimulation	Object naming, reading, recall of non-words, verbal working memory, −100	rTMS over STG: ↓correctness in reading, ↓correctness in recall of non-words; rTMS over MTG: ↓RTs in object naming
Schuhmann et al. (2012)	12 HY	tpTMS (40 Hz, 50 ms), 120% of MT	WA, left MTG, BA, placebo, no stimulation	Naming of objects, +150/+525	rTMS over MTG at 225 and 400 ms: ↑RTs rTMS over BA at 300 ms: ↑RTs rTMS over WA at 400 ms: ↑RTs
Cotelli et al. (2012)	10 PNFA, 4 SD	On-line rTMS (20 Hz, 500 ms), 90% of MT	Right DLPFC, left DLPFC, placebo	Naming of objects and actions, 0	TMS over left and right DLPFC: ↑ correctness for actions in PNFA
TRANSCRANIAL DIRECT CURRENT STIMULATION					
Sparing et al. (2008)	15 HY	atDCS, ctDCS (2 mA, reference Cz), placebo	WA, WAH, placebo	Object naming	atDCS over WA: ↓RTs
Fertonani et al. (2010)	12 HY	atDCS, ctDCS (2 mA, reference shoulder), placebo	Left DLPFC	Object naming	atDCS over left DLPFC: ↓RTs
Holland et al. (2011)	10 HE	atDCS (2 mA, reference contralateral area), placebo	Left IFC	Object naming	atDCS over left frontal cortex: ↓RTs, decreased activity in Broca's area
Wirth et al. (2011)	20 HY	atDCS (1.5 mA, reference shoulder), placebo	Left DLPFC	Object naming (semantically related or unrelated blocks)	atDCS over left DLPFC: trend of ↓RTs

*N, number of participants; MT, motor threshold; SO, stimulator output; DLPFC, dorsolateral prefrontal cortex; IFC, inferior frontal cortex; MTG, middle temporal gyrus; STG, superior temporal gyrus; BA, Broca's area; WA, Wernicke's area; WAH, WA Homologous; TMS, transcranial magnetic stimulation; spTMS, single pulse TMS; tpTMS, three pulses TMS; rTMS, repetitive transcranial magnetic stimulation; atDCS, anodal transcranial direct current stimulation; ctDCS, cathodal transcranial direct current stimulation*.

**Figure 3:**
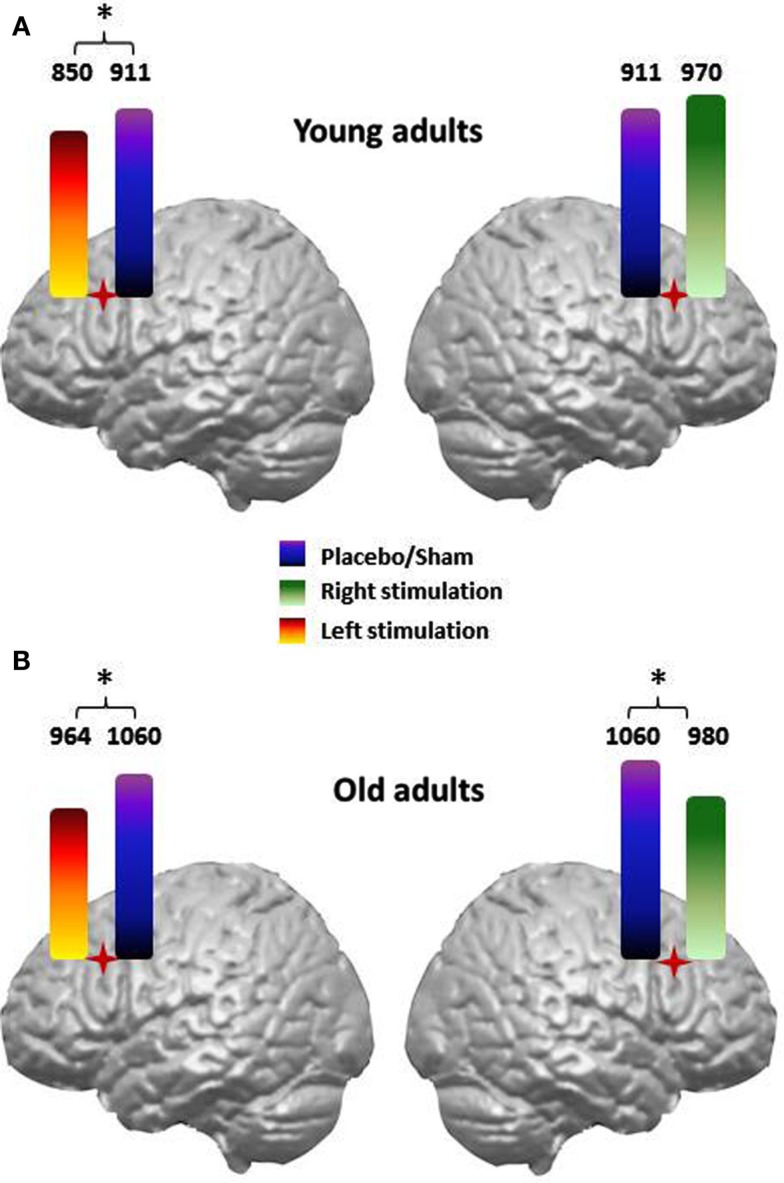
**Verbal reaction times (vRTs) achieved during an action naming task in younger (A) and older adults (B) following right and left dorsolateral prefrontal cortex (DLPFC) stimulation when compared to sham/placebo stimulation**. The facilitatory effect induced by left or right DLPFC stimulation is plotted separately for action and object stimuli. In the older participants, the vRTs for actions were consistently faster during real left or right DLPFC stimulation than during sham stimulation. No significant differences between real and sham stimulation were observed for object naming.

The involvement of the dorsolateral prefrontal cortex (DLPFC) in naming tasks has also been evaluated. Cappa et al. (2002) reported selective facilitation of action (but not object) naming when young adults received rTMS to the left DLPFC; the verbal reaction times (vRTs) were shortened following rTMS compared to sham (i.e., placebo) stimulation. A unique study investigated action and object naming in older adults (Cotelli et al., 2010) by applying rTMS over the left or right DLPFC. Cotelli et al. (2010) observed that although the rTMS effect in younger adults (Cappa et al., 2002) was limited to left-sided stimulation, facilitation in older adults was bilateral, which is demonstrated in Figure 3.

Functional neuroimaging studies have revealed that older adults tend to recruit regions of the contralateral hemisphere in addition to regions of the specialized hemisphere when performing cognitive tasks (Grady et al., 1998; D'Esposito et al., 1999; Grady, 2000; Rypma and D'Esposito, 2000; Cabeza et al., 2002; Logan et al., 2002; Stebbins et al., 2002; Wierenga et al., 2008). According to the frontal compensation hypothesis, the presence of a facilitation effect following right or left DLPFC stimulation in older adults may be attributed to the presence of a compensatory mechanism based on the recruitment of right hemispheric resources to maintain task performance despite the possible reduction in neural efficacy of a distributed naming network. A shift from unilateral to bilateral engagement is consistent with Cabeza's (Cabeza, 2002; Cabeza et al., 2002) model of hemispheric asymmetry reduction in older adults (HAROLD). The HAROLD hypothesis proposes that bilateral activation increases with age. Although evidence from many cognitive tasks supports the HAROLD hypothesis (Grady et al., 1995; Rossi et al., 2001; Cabeza, 2002), the importance of these lateralization reductions remains unclear. Only one NIBS study (Manenti et al., 2011) that tested episodic memory abilities in elderly individuals supports the frontal compensation hypothesis. Manenti et al. (2011) highlighted the presence of a reduction in asymmetry that was specific to older adults who performed better on memory tests (similar to younger adults) in comparison to older adults whose performances were worse on memory tests (for a review, see Manenti et al., 2012).

Cotelli et al. (2010) demonstrated that both right and left DLPFC stimulation during action naming led to shorter vRTs (Figure 3) and suggested that these data provide evidence of aging-associated cortical plasticity. The right hemisphere becomes functionally relevant during naming to compensate for the aging-related decreased efficiency of the left hemisphere. Further studies are needed to more thoroughly understand the functional significance of this bilateral recruitment.

Cotelli and collaborators (Cotelli et al., 2006b; Miniussi and Rossini, 2011) applied rTMS in AD patients and found that stimulation of the DLPFC improves naming performance in AD patients at both early (Cotelli et al., 2006b) and advanced stages (Cotelli et al., 2008) of cognitive decline. Compared to the sham stimulation condition, the correctness scores of the AD patients increased following left and right rTMS to DLPFC. Moreover, in patients in the early stages of cognitive decline, rTMS improved action naming performance only, while rTMS improved both action- and object-naming performance in patients at more advanced stages of cognitive decline.

Although several studies have been conducted using TMS during naming tasks, only three studies have used tDCS to study naming processes in younger or older adults. Sparing et al. (2008) verified the role of Wernicke's area in object naming by demonstrating that anodal tDCS over this area induced shorter vRTs in young adults. The other two studies involving young adult participants demonstrated the DLPFC's role during naming, revealing shorter vRTs during anodal tDCS compared to cathodal tDCS or placebo stimulation (Fertonani et al., 2010; Wirth et al., 2011). A unique fMRI-tDCS study investigated the role of the inferior frontal cortex during object naming in older adults (Holland et al., 2011) and demonstrated that the left inferior frontal cortex is critical to naming ability; vRTs were shortened during anodal tDCS compared to during the placebo condition. Furthermore, faster naming responses correlated with a decreased blood-oxygen-level-dependent signal in Broca's area, which reinforced the importance of this region within the normal naming network.

In summary, several NIBS studies have highlighted frontal and temporal areas that are causally involved in naming in younger adults, whereas only two studies have investigated naming in older adults. A few studies have utilized this technique to examine age-related pathologies and have demonstrated increased performance following DLPFC stimulation. Overall, these results suggest that it would be interesting to use NIBS to modulate performance, to study functional adaptation in physiological aging and eventually to attempt to reduce cognitive deficits in pathological brain aging.

## Conclusion

In this article, we reviewed studies that utilized NIBS to study language processing in younger and older adults and in dementia patients. In particular, we focused on a study of naming in older participants using TMS (Cotelli et al., 2010). The age-related changes that were observed in this TMS naming study are consistent with other neuroimaging discoveries and theories of cognitive aging. This study underlines the presence of a facilitatory effect on naming following right or left DLPFC stimulation in older adults; this result is in contrast to the unilateral (i.e., left) effect that was previously observed in young adults. This bilateral frontal effect may be attributed to the presence of a compensatory mechanism that is based on the recruitment of right hemisphere resources to maintain task performance (Cabeza, 2002; Cabeza et al., 2002). The same mechanisms could underlie the increased naming accuracy induced by both left and right DLPFC TMS in neurodegenerative patients (Cotelli et al., 2006b, 2008, 2012).

## Future Directions

Because NIBS techniques were shown to modify cortical activity, these methods may assist in the evaluation of naming functional changes at the cortical level in aging subjects. Based on its facilitatory behavioral effects in normal and pathological subjects, NIBS could also be used to facilitate “scaffolding” (the manifestation of compensatory networks) in all types of individuals who experience naming difficulties.

## Conflict of Interest Statement

The authors declare that the research was conducted in the absence of any commercial or financial relationships that could be construed as a potential conflict of interest.

## Key Concept

Age-related brain changes: The studies of physiological and pathological brain aging achieved over the last two decades has provided strong evidence of neurophysiological correlates of cognitive and behavioral changes associated with aging.
Neural strategies: The STAC presents a reunified vision of the dynamic brain changes that occur in response to naturally occurring functional alterations across the life span (Park and Reuter-Lorenz, 2009).
Age-related decline in object and action naming: Several studies have demonstrated an age-related decline in object and action naming suggesting that word retrieval declines in healthy aging and in pathological aging.
Non-Invasive Brain Stimulation (NIBS): Non-invasive brain stimulation techniques for modulating cortical activity include TMS and tDCS. Both TMS and tDCS can transiently influence behavior by altering spontaneous neuronal activity, which may have facilitatory or inhibitory effects.: This review underlines the presence of a facilitatory effect on naming following right or left DLPFC rTMS stimulation in older adults. This bilateral frontal effect may be attributed to the presence of a compensatory mechanism that is based on the recruitment of right hemisphere resources to maintain task performance.
